# Evaluating the Integrated Plastic Surgery Residency Match During the Novel Coronavirus Pandemic

**DOI:** 10.7759/cureus.16988

**Published:** 2021-08-07

**Authors:** Cees T Whisonant, Shawhin R Shahriari, Joshua Harrison, Amanda Ederle, Samantha J Marley, Hannah E Dowdy-Sue, Gregory Borah

**Affiliations:** 1 Division of Plastic, Reconstructive, Hand and Burn Surgery, Department of Surgery, University of New Mexico School of Medicine, Albuquerque, USA; 2 Internal Medicine, Baptist Health, Little Rock, USA

**Keywords:** plastic and reconstructive surgery, integrated plastic surgery, covid 19, medical residency, the match

## Abstract

The COVID-19 pandemic had significant impacts on medical education and on the 2021 Match. Visiting student rotations at locations other than students’ home institutions were cancelled and residency interviews were hosted virtually. This study evaluated the impact that COVID-19 had on the 2021 Match including residency programs matching applicants from within their own institution as well as from within the same region. The sex of matched applicants in the Match cycles was analyzed as well. Data were collected from residency program websites, social media accounts, and communication with current residents. Data were tabulated and chi-square analysis was performed. The overall difference in matched internal candidates pre-/post-pandemic was determined to be statistically significant (8.3% increase; p = 0.004). The Midwest was determined to exhibit a significant increase for matching residents from medical schools in the same region (15.6% increase; p = 0.04). Female applicants were also determined to be significantly more likely to match into integrated plastic surgery programs in 2021. COVID-19 significantly impacted the 2021 Match with an increased number of programs selecting internal candidates, matched female applicants, and regional selectivity, especially in the Midwest. It is our hope that applicants, programs, and the plastic surgery community will use this information to continue to improve the residency selection process in the future.

## Introduction

Medical students and American College of Graduate Medical Education (ACGME) accredited residency community members navigate the process of the National Residency Matching Program (NRMP) Match annually. There have been multiple studies that previously looked at the NRMP Match within plastic surgery including program director questionnaires [1,2] and studies reviewing characteristics of individuals who successfully match to a plastic surgery program [3-7]. Applying broadly is a common practice in plastic surgery, and other competitive specialties, and imposes a significant financial burden upon applicants [3]. This includes the cost of the application itself as well as travel and lodging during interviews, the latter of which was not a factor during the 2021 Match. Time is another essential resource that applicants expend when applying to and interviewing at programs across the country. These are only a few of the factors that go into the Match process, though the members of the field are continually trying to demystify what application components lead to a successful plastic surgery match.

The COVID-19 pandemic has impacted many lives in a variety of ways, including the lives of students preparing for the residency application process. In the wake of the COVID-19 pandemic, fourth-year medical students were unable to complete rotations outside their own institutions except for students that did not have a specific program of interest at their home institution [8]. This drastically changed the dynamic of the application process, especially in a field like plastic surgery, where away rotations are vital to a successful match [2]. Additionally, another major, unexpected change 2021 applicants faced was virtual interviewing rather than in-person interviews where applicants traditionally have the opportunity to experience a program first-hand [8].

Given these changes and the highly competitive nature of the plastic surgery residency Match under normal circumstances, we hypothesized that an applicant would be more likely to match at their home program during the 2021 Match cycle (i.e., where the applicant went to medical school) compared to previous years. We reviewed the NRMP Match results to determine if there was an internal applicant preference or regional preference comparing the 2021 results to previous Match cycles from 2016 to 2020.

## Materials and methods

Authors used the ACGME website to determine accredited plastic surgery residency programs during the 2016-2021 application cycles and the number of residency spots available each year per program [9]. Data were obtained from integrated plastic surgery program websites and social media accounts including Instagram, Facebook, and Twitter. The data included matched applicants for each program and the medical school they attended.

This information was analyzed to determine if there was a change in the trends from previous years when comparing the 2021 NRMP Match versus 2016 to 2020. The data were tabulated and analyzed utilizing chi-square analysis of pre-COVID-19 Match years versus the COVID-19 Match year (i.e., 2016 - 2020 vs. 2021). A p-value of less than 0.05 was considered statistically significant.

## Results

2021 versus previous cycles overall

Between 2016 and 2021, there were 15 new ACGME accredited integrated plastic surgery residency programs (Table 1) which was a 21.7% increase [10,11]. There was a total of 34 first-year plastic surgery residency positions added between 2016 and 2021, amounting to a 22% increase. In 2021, there were two new integrated plastic surgery programs and seven more residency positions added.

**Table 1:** Integrated plastic surgery residency programs and positions 2016-2021

Year	Number of residency programs	Number of residency positions
2016	69	153
2017	73	163
2018	77	172
2019	78	174
2020	82	180
2021	84	187

Internal candidates 

When reviewing pre-COVID-19 Match data from 2016 to 2020 (Table 2), 16.6% of successful matches were found to be internal applicants. In 2021, 24.9% of spots were filled by internal candidates.

**Table 2:** Internal applicants selected 2016-2021

	2016	2017	2018	2019	2020	2021
Internal candidates	26 (17.0%)	34 (20.9%)	28 (16.3%)	24 (13.8%)	29 (16.1%)	48 (25.9%)

The overall difference between pre-COVID-19 versus COVID-19 internal candidate match rates (Table 3) was found to be statistically significant (p = 0.004). The programs that were most likely to match their internal applicants from 2016 to 2020 were Duke University (53.3%), University of Texas Medical Branch Hospitals (35.0%), and Washington University in St. Louis (33.3%).

**Table 3:** Pre-COVID-19 and COVID-19 Match year statistics for a region, home institution, and sex

	Pre-COVID-19 (2016-2020)	COVID-19 (2021)	p-value
Region			
Midwest	17 (39.5%)	27 (55.1%)	0.04
Northeast	22 (46.9%)	31 (58.5%)	0.13
South	24 (54.1%)	31 (63.3%)	0.31
West	10 (30.8%)	15 (44.1%)	0.08
Residency Program/Medical School			
Same	28 (16.6%)	48 (25.9%)	0.004
Sex			
Male	94 (55.8%)	81 (44%)	0.004
Female	74 (44.2%)	103 (66%)	

In 2021, the institution that matched the most internal applicants was Georgetown University with three of four residency spots filled by graduates of Georgetown. Schools that matched multiple internal candidates in 2021 are shown in Table 4. Of note, 13 programs that had previously not taken internal applicants between 2016 and 2020 accepted first-year residents from their own institution in 2021.

**Table 4:** Residency program positions filled by multiple internal candidates in 2021

School	Total positions	Positions filled by internal candidates
Georgetown University	4	3
New York University	4	2
University of Michigan	4	2
University of Pennsylvania	3	2
University of Wisconsin	3	2
University of California - Irvine	3	2
Northwestern University	2	2
Louisiana State University	2	2
University of Miami	2	2

Regional selection 

From 2016 to 2020, successful applicants to residency programs from the same region as their medical school (Table 5) were found to make up on average 44.0% of total residents. The region with the most residents from medical schools in the same region was the South (54.1%). An increase was seen in preferential regional selectivity in 2021 with 56.2% of matched applicants at residency programs coming from a medical school of the same region. An increase was seen across all regions individually as well in terms of region selectivity with the largest increase, and an only statistically significant increase from past years, occurring in the Midwest (15.6% increase; p = 0.04).

**Table 5:** Same US region medical school and residency program selection *Hashmi et al. [12].

	2015*	2016	2017	2018	2019	2020	2021
Midwest	42.2%	17 (41.5%)	17 (38.6%)	18 (40.0%)	17 (37.0%)	19 (40.4%)	27 (55.1%)
Northeast	57.7%	21 (48.8%)	17 (38.6%)	22 (44.9%)	30 (58.8%)	22 (43.1%)	31 (58.5%)
South	50.0%	8 (21.1%)	20 (45.5%)	30 (66.7%)	32 (72.7%)	31 (64.6%)	31 (63.3%)
West	13.2%	8 (26.7%)	7 (22.6%)	9 (27.3%)	12 (36.4%)	14 (41.2%)	15 (44.1%)
Total		54 (35.5%)	61 (37.4%)	79 (45.9%)	91 (52.3%)	86 (47.8%)	104 (56.2%)

## Discussion

Previous studies relating to the Match in plastic surgery have included multiple program director questionnaires regarding applicant characteristics, standardized test scores, Alpha Omega Alpha Society (AOA) membership, and even regional bias in selecting residents [1,2,4-7,12]. Resident selection is a multifactorial process that has many unknowns, therefore, reviewing data from previous application years is valuable in analyzing the factors that lead to successful matches. Improving the selection process and demystifying these factors are valuable for both residency programs and applicants in order to allow the most qualified individuals the best chance to match into the competitive specialty of plastic surgery.

Internal matching has been previously studied and has demonstrated that 19.6% of medical students match at their home institution [12]. We found that 24.9% of students in the 2021 cycle matched at their home institution, compared to the 16.6% that matched previously in cycles from 2016 to 2020. Factors that contributed to this increase in internal matching could include lack of away rotations, limited perception of outside plastic surgery programs, and the online interface of 2021 interviews [2,12,13]. We found that larger programs were more likely to match students from their home institution which was consistent with the findings of Hashmi et al. [12].

Both residency directors and applicants alike reported that “making a good impression” or finding a “good fit” was their primary goal for away rotations. Drolet et al. found that 91.1% of plastic surgery applicant respondents felt that their away rotations shaped them into stronger applicants, and program directors reported that a strong away rotation was the most important residency selection criterion when selecting an applicant [2]. Perceptions of plastic surgery programs across the nation were limited in 2021 due to travel restrictions and the inability of students to experience a program first-hand. Because interviews were virtual and away rotations were limited, these important factors were unable to be assessed at the applicant’s potential residency program interviews. However, students were still able to view their home program and interact with the faculty and residents at their school, making this an obvious choice. Because students and program directors had more limited face-to-face contact and exposure limited to the virtual atmosphere, they were faced with making a difficult decision. These variables are some of the possible reasons for the increase in home-institution matching in 2021.

In terms of regionality, previous studies by Hashmi et al. have demonstrated a bias for residency programs accepting applicants who come from a medical school in the same region. Specifically, they noted that programs in the Northeast tended to match residents from the same region, at the highest percentage (57.7%) [12]. We compared the past 2016-2020 data with Hashimi’s 2015 data and with the 2021 (COVID-19) Match data (Table 5). In reviewing the data from 2016 to 2021, there does seem to be a regional bias; however, it appears to be residency programs in the South who select applicants from their own region at the highest percentage both pre-COVID-19 years as well as the COVID-19 Match (54.1% and 63.3%, respectively). Programs in other regions were found to match a high percentage of residents from their own region at a high percentage as well between 2016 and 2020. This regional selection seems to have increased for all regions in 2021, where more programs matched individuals from the same region. In 2021, successfully matched applicants were found to have attended medical school in the same region as their residency training programs at an increased percentage for all regions.

When comparing 2021 to previous years, our data indicate that there was not only an increased selection of internal applicants but also an increased preference for residents within the same region. There were no away rotations during the 2020-2021 cycle, except in situations in which applicants did not have a home plastic surgery program. These applicants were recommended to rotate at a program within their region [8]. As hypothesized, COVID-19 has changed the Match, in that there was an increased internal and regional preference.

As shown in Table 1, from 2016 to 2021 there was found to be an increase of ACGME accredited integrated plastic surgery residency training programs (15) and total residency positions nationally (34) [9]. During this time period, we noted an increase in the number of female residents (Table 6), with 62 (40.8%) matched applicants being female in 2016 compared to 84 (46.7%) in 2020. Although the percentage of matched applicants that were male has gone down during this time period, the total number of matched male applicants remained nearly the same annually. An interesting change in terms of the gender composition of incoming first-year residents in plastic surgery occurred in 2021 with 81 males (44.0%) successfully matching compared to 103 females (56.0%). Given the gender gap in plastic surgery (and all surgical subspecialties), it is reassuring to see the trend of more female successful matches. It is still unclear what factors, applicant or residency program-related, have led to certain programs being more likely to match female residents; this would be a valuable area of further investigation.

**Table 6:** Residency positions filled by males and females 2016-2021

	2016	2017	2018	2019	2020	2021
Male	90 (59.2%)	95 (58.6%)	95 (55.2%)	93 (53.4%)	96 (53.3%)	81 (44.0%)
Female	62 (40.8%)	67 (41.4%)	77 (44.8%)	81 (46.6%)	84 (46.7%)	103 (66.0%)

Limitations of our study are due to the data collection process which used a search of different program’s websites, social media accounts, and word of mouth. Additionally, this study does not account for back-filled spots, including when a program becomes newly accredited or if their resident complement is increased. Other confounding factors include residents who performed research years at specific institutions, any away rotations they may have done as applicants, along the multitude of additional factors that go into the residency application process. This study did not assess the impact of the pandemic for those without home programs or regions to match into such as osteopathic medical school graduates or international medical graduates. These would be interesting areas for further research. 

The Match system is anticipated to be affected by the change of the USMLE Step 1 examination scoring to pass/fail and will be a challenge for those involved in the residency selection process. It is unclear what factors will become more important in terms of resident selection following this change as this examination is one of the few standardized objective measures across all medical schools. Previously, Boyd et al. theorized that research productivity may become increasingly important for applicant success, similar to what occurred in Canada after implementing a pass/fail system for their national board examination [14,15]. It will be interesting to see how residency program directors will respond to these changes across all fields of medicine, especially in competitive specialties such as integrated plastic surgery. 

## Conclusions

The COVID-19 pandemic has complicated the annual residency selection process in new and challenging ways. Plastic surgery training programs have tried to account for these changes in the hopes that a holistic view can be taken of applicants while simultaneously preventing those who have the potential to be excellent plastic surgeons from falling through the cracks. The COVID-19 pandemic clearly impacted the plastic surgery residency Match as the percentage of internal candidates selected was shown to increase significantly. With the uncertainty that surrounds the coming Match cycles following the COVID-19 pandemic, these results are particularly valuable to applicants and program directors. As interviews may continue to be virtual and away rotations limited, it is unlikely the Match in 2022 will differ significantly from 2021. If this is the case, applicants would be well advised that their best opportunity to successfully match is within their home region, especially at the program at which they attended medical school.
